# Ammonia gas sensors based on chemically reduced graphene oxide sheets self-assembled on Au electrodes

**DOI:** 10.1186/1556-276X-9-251

**Published:** 2014-05-21

**Authors:** Yanyan Wang, Liling Zhang, Nantao Hu, Ying Wang, Yafei Zhang, Zhihua Zhou, Yanhua Liu, Su Shen, Changsi Peng

**Affiliations:** 1 College of Physics, Optoelectronics and Energy, Collaborative Innovation Center of Suzhou Nano Science and Technology, Soochow University, Suzhou 215006, People's Republic of China; 2 Key Laboratory for Thin Film and Micro fabrication of the Ministry of Education, Department of Microelectronics and Nanoscience, School of electronic information and electrical engineering, Shanghai Jiao Tong University, Shanghai 200240, People's Republic of China; 3 State Key Laboratory of Electronic Thin Films and Integrated Devices, School of Microelectronics and Solid-State Electronics, University of Electronic Science and Technology of China, Chengdu 610054, People's Republic of China

**Keywords:** Graphene, Self-assembly, Pyrrole, Ammonia, Gas sensor

## Abstract

We present a useful ammonia gas sensor based on chemically reduced graphene oxide (rGO) sheets by self-assembly technique to create conductive networks between parallel Au electrodes. Negative graphene oxide (GO) sheets with large sizes (>10 μm) can be easily electrostatically attracted onto positive Au electrodes modified with cysteamine hydrochloride in aqueous solution. The assembled GO sheets on Au electrodes can be directly reduced into rGO sheets by hydrazine or pyrrole vapor and consequently provide the sensing devices based on self-assembled rGO sheets. Preliminary results, which have been presented on the detection of ammonia (NH_3_) gas using this facile and scalable fabrication method for practical devices, suggest that pyrrole-vapor-reduced rGO exhibits much better (more than 2.7 times with the concentration of NH_3_ at 50 ppm) response to NH_3_ than that of rGO reduced from hydrazine vapor. Furthermore, this novel gas sensor based on rGO reduced from pyrrole shows excellent responsive repeatability to NH_3_. Overall, the facile electrostatic self-assembly technique in aqueous solution facilitates device fabrication, the resultant self-assembled rGO-based sensing devices, with miniature, low-cost portable characteristics and outstanding sensing performances, which can ensure potential application in gas sensing fields.

## Background

Chemiresistive sensors have aroused much attention in environment monitoring, industry and agriculture production, medical diagnosis, military, and public safety, etc. nowadays [1-5]. In order to meet the requirements of industry and other fields' demands, semi-conducting metal oxide, organic semiconductors, and carbon materials, etc., which have high aspect ratio and large specific surface area, have been widely used as sensing materials and the excellent performances of the resultant devices have been achieved [6-8].

Graphene, as a new member of carbon family, has emerged as a promising candidate for sensing because of its unique electronic, excellent mechanical, chemical, and thermal properties [9-18]. Excellent sensing performance of graphene towards different kinds of gases, including NO_2_, NH_3_, H_2_O, CO, trimethylamine, I_2_, ethanol, HCN, dimethyl methylphosphonate (DMMP), and DNT, have been reported [19-26]. Generally, there are three main methods to prepare graphene materials: micromechanical exfoliation of graphite [16], chemical vapor deposition [27], and reduction of graphene oxide (GO) [28]. The resultant graphene materials can be considered as excellent candidates for gas sensing, especially for chemically reduced graphene oxide (rGO). The rGO sheets have great potential for using as chemiresistors [29-32] due to their scalable production, easy processability in solution, large available surface area, etc. Hydrazine and ascorbic acid have been reported as excellent reducing agents for the reduction of GO, and the resultant rGO sheets show excellent responses to different vapors [20,33]. Although many reports have been reported on the rGO sensing devices, it is still a great challenge to develop chemiresistive sensors based on rGO with miniature, low-cost, and portable characteristics.

In order to fabricate chemiresistive sensors based on nanomaterial, there are generally two main methods. One is to deposit nanomaterial on substrates followed by patterning electrodes on top of sensing materials [34]. However, the process is complicated and requires exquisite skills. The other fascinating method is to drop-cast nanomaterial solution onto the pre-patterned electrode surfaces [29,35]. This technique is facile, less expensive with higher yields, since it can be operated in solution, which benefits for the large-scale fabrication of the sensing devices. However, drop-casting method is very hard to ensure the reproducibility of the fabricated devices, which needs to be improved and applied in the realistic detection fields.

Herein, we report a facile and controllable self-assembly technique to fabricate rGO sensors, which could be used as an excellent NH_3_ gas sensing device. Negative GO sheets with large sizes (>10 μm) can be easily electrostatically attracted onto positive Au electrodes modified with cysteamine hydrochloride in aqueous solution. The assembled GO sheets on Au electrodes can be directly reduced into rGO sheets by hadrazine or pyrrole vapor and consequently provide the sensing devices based on self-assembled rGO sheets. In addition, pyrrole-vapor-reduced rGO-based sensor exhibits excellent response to NH_3_. We expect the easy, reproducible, green, and scalable fabrication of the sensors based on rGO reduced by pyrrole, with excellent performance, miniature, low-cost, and portable characteristics, can pave a new avenue for the application of assembled rGO devices in gas sensing field.

## Methods

### Materials

The natural graphite (32 meshes) used in this study was obtained from Qingdao Jinrilai Co. Ltd, Qingdao, China. Pyrrole was obtained from Shanghai Chemical Reagents Co. Ltd. (Shanghai, China) and purified by distillation. Pre-determined NH_3_ gas (1 ppm) mixed with air was purchased from Beijing Beiyang Special Gases Institute Co. Ltd. (Beijing, China). Concentrated ammonia solution (25 wt.%) and all of other chemicals (analytical reagent grade) were purchased from Shanghai Chemical Reagents Co. Ltd. (Shanghai, China) and were used without further purification. All of organic solvents were purified by distillation.

### Self-assembly of GO sheets on Au electrodes

GO sheets with large sizes were prepared similar to the method reported by Zhao et al. [36]. Large-size GO aqueous solution with the concentration at 2.5 mg/mL was prepared by mild sonication (80 W for 5 min) and stored for the further self-assembly process.

The standard microfabrication procedures were exploited to obtain the Au electrodes according to the method illustrated by us before [37]. The parallel electrodes with the gap distance of 1 μm were formed by sputtering 10 nm Cr and 180 nm Au onto a patterned photoresist mold. A lift-off process was further carried out to remove the photoresist. The resultant electrodes were sonicated in ethanol, washed with deionized water thoroughly, and finally dried by nitrogen flow.

In order to obtain positively charged Au electrodes, the electrodes were immersed in 1 mM of cysteamine hydrochloride aqueous solution for 24 h, followed by washing with water and ethanol successively, each for three times. The resultant positive electrodes were further immersed in GO aqueous solution with different concentrations (1, 0.5, and 0.25 mg/mL) for 24 h. After washing with water and ethanol, each for three times, the electrodes were dried by purging air. Consequently, GO sheets bridged between Au electrodes were fabricated.

### Chemical reduction of assembled GO sheets on Au electrodes

The GO sheets on the electrodes were easily reduced by hydrazine or pyrrole vapor. Typically, the electrodes with GO sheets were put in a vessel, and 3 drops of hydrazine were added besides the electrode. Then the vessel was sealed and put into the oven with the temperature at 90°C for 12 h. The resultant rGO sheets on the electrodes, denoted as Hy-rGO, were washed with distilled water and ethanol (each for three times) and dried by purging air.

For the purpose of the comparison, the rGO reduced by 3 drops of pyrrole, denoted as Py-rGO, was also fabricated according to the method mentioned above.

### Characterizations

Atomic force microscope (AFM) was performed using a Dimension Icon instrument (Veeco, Plainview, New York, USA). The morphologies of the samples on the electrodes were observed by field emission scanning electron microscopy (FE-SEM; Carl Zeiss Ultra 55, Carl Zeiss AG, Oberkochen, Germany). Raman scattering was performed on a Renishaw inVia Reflex Raman spectrometer (Renishaw, Zhabei District, Shanghai, China) using a 514-nm laser source.

The sensing tests were carried out on a homemade gas handling system as illustrated in our previous report [35]. The NH_3_ environments with the concentrations at parts per billion and parts per million levels were easily produced by diluting the NH_3_ gas with dry air. The humidity inside the test chamber was monitored by a Honeywell HIH-4000 humidity sensor (Honeywell Inc., Shanghai, China) and less than 5%. All of the sensing tests were carried out using a precision semiconductor parameter analyzer (Agilent 4156C; Agilent Technologies, Beijing, China) at room temperature. The flow rate of the balance gas (dry air) was controlled to be at 1 L/min. The sensor response was evaluated by the resistance change at a sampling voltage of 50 mV.

## Results and discussion

### Self-assembly technique for the fabrication of devices based on rGO sheets

**Figure 1:**
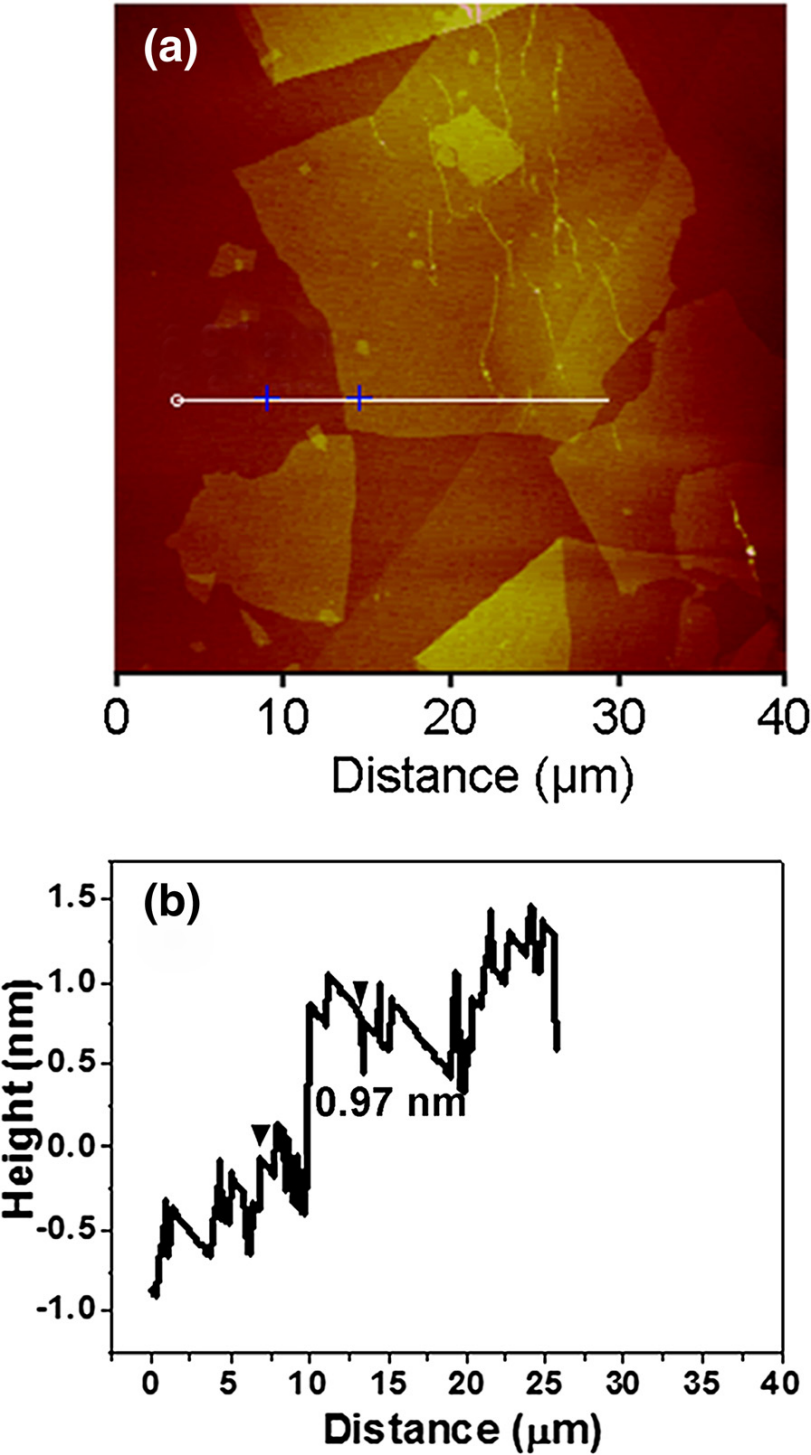
AFM image (a) and height profile (b) of GO sheets deposited on mica surfaces.

In order to make sure the rGO sheets bridge the gaps of the parallel Au electrodes, GO sheets with large sizes were prepared in this work. Natural graphites with large sizes (32 meshes) were used as original materials, and a modified Hummers method was exploited similar with the work reported by Zhao et al. [36]. Dialysis was carried out for the purpose of complete removal of acid in the suspension, and mild sonication was applied in order to avoid the destruction of GO sheets. As a result, single GO sheets were formed in aqueous solution and large sizes were maintained as well. The morphology of GO sheets was observed by AFM; the results were shown in Figure 1. As shown in Figure 1a, the sizes of the majority of GO sheets were larger than 10 μm, which was in consistence with the results of SEM images of electrodes discussed later. Furthermore, the height profile of the AFM image (Figure 1b) indicated that the thickness of the obtained GO sheet was about 0.97 nm, suggesting the successful achievement of the single-layer GO sheets [38]. As we know, GO sheets contain a large number of negative functional groups (e.g., hydroxyl and carboxyl groups) [39], which can be a benefit for their electrostatic attraction with positive surfaces during the self-assembly process.

**Figure 2:**
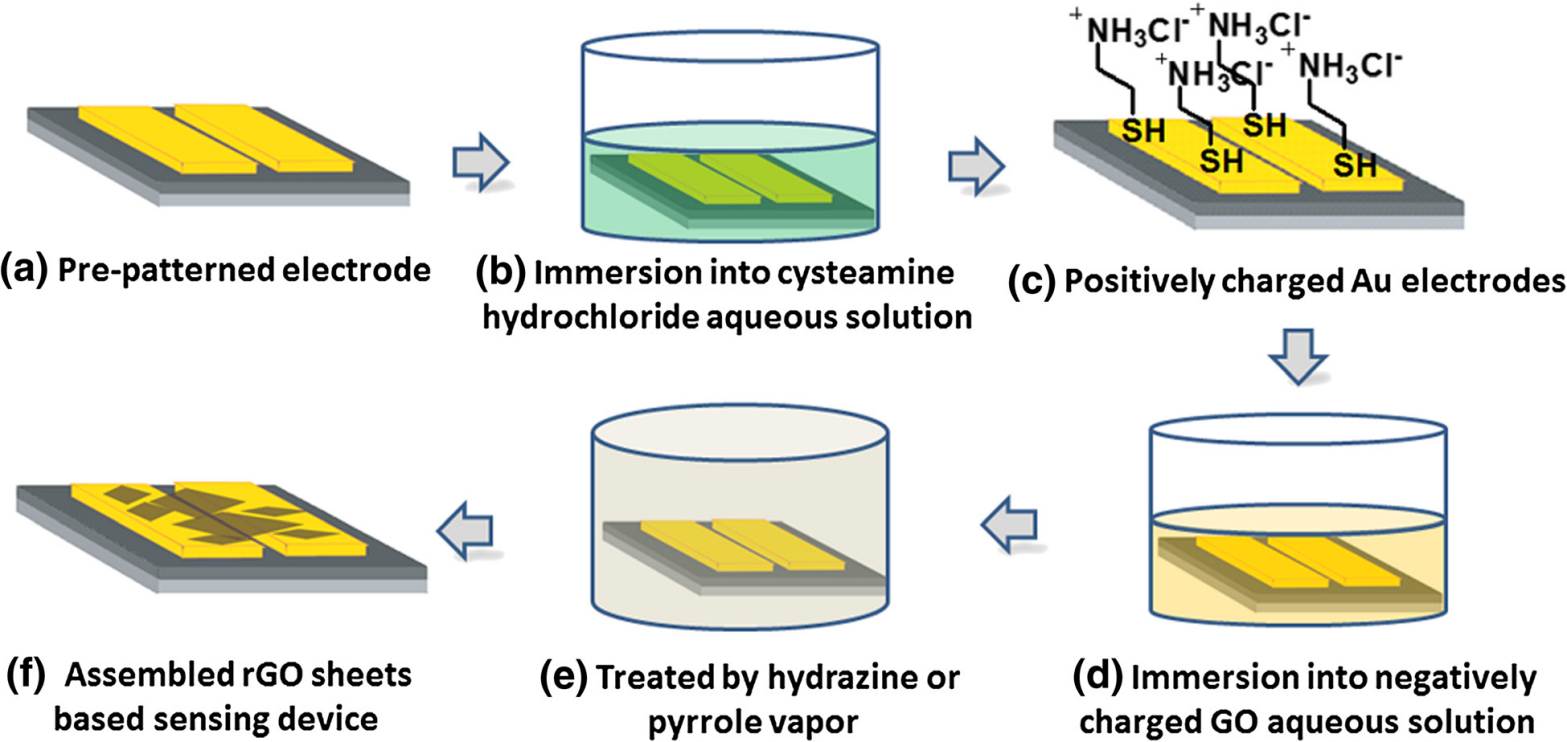
Schematic illustration of the fabrication of sensing devices based on self-assembled rGO sheets.

The sensing devices were fabricated by self-assembly of the obtained GO sheets on Au electrodes, followed by *in situ* reduction by hydrazine or pyrrole vapor. The process was schematically illustrated in Figure 2. The parallel Au electrodes on SiO_2_ (300 nm)/Si wafers were easily patterned by a standard microfabrication process, and the distance of the gap was fixed at about 1 μm in order to make sure GO sheets be easily bridged on between paralleled Au electrodes. Since electrostatic attraction was applied as driving forces for self-assembly of negative GO sheets on Au electrodes, Au electrodes were treated by cysteamine hydrochloride aqueous solution in advance to attach positively charged amine groups. As we know, organic molecules with thiol groups can be assembled on the surface of Au through forming self-assembled monolayers (SAMs) due to the strong affinity between sulfur and Au [40,41]. Hence, SAMs with positively charged amine groups on the surface of Au electrodes were formed during this assembly process. The resultant Au electrodes assembled with GO sheets were further put in sealed vessels and reduced by hydrazine or pyrrole vapor at 90°C; the GO sheets on Au electrodes were *in situ* reduced into rGO and consequently formed the sensing devices based on assembled rGO sheets.

**Figure 3:**
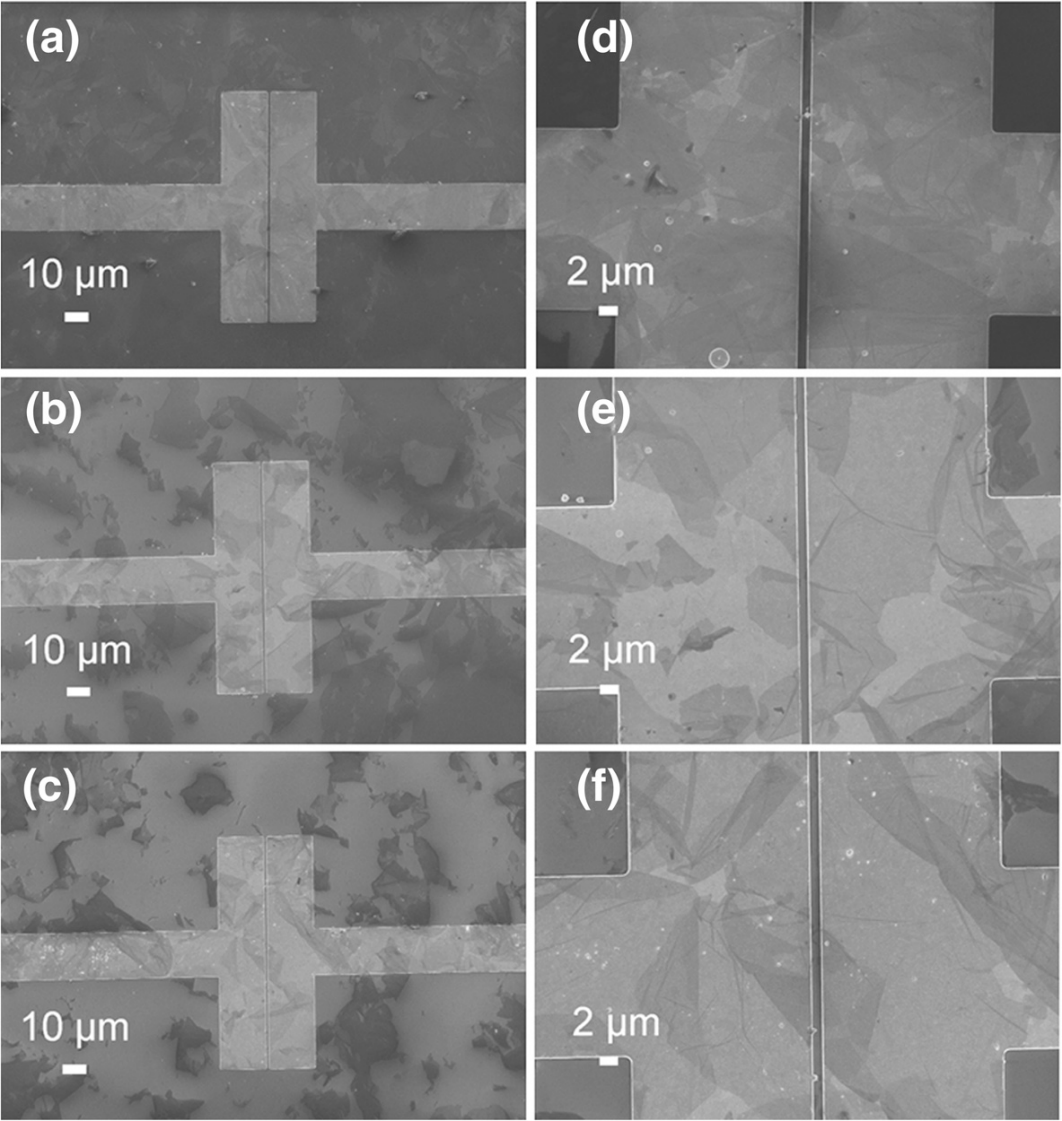
**SEM images of GO sheets bridged between Au electrodes self-assembled with different concentrations of GO. (a)** and **(d)** 1 mg/mL, **(b)** and **(e)** 0.5 mg/mL, and **(c)** and **(f)** 0.25 mg/mL.

Figure 3 shows SEM images of GO sheets bridged between Au electrodes self-assembled with different concentrations of GO sheets. GO aqueous solutions, with different concentrations (1, 0.5, and 0.25 mg/mL), were used to assemble on between Au electrodes. The morphologies of the resultant Au electrodes with GO sheets were shown in Figure 3a, b, c, d, e, f. From Figure 3, we can observe that the sizes of the majority of GO sheets were larger than 10 μm, which was in agreement with AFM results. Furthermore, when the concentration of GO solution was as high as 1 mg/mL, a thick layer of GO sheets were formed on Au electrodes (as shown in Figure 3a, d). As the concentration of GO solution decreases, fewer GO sheets on the Au electrodes were observed (as shown in Figure 3b, c, e, f). Moreover, from the enlarged images (Figure 3e, f), we can observe that GO sheets bridged between Au electrodes have been successfully formed. The morphologies of electrodes assembled with lower GO concentration were not given here, since further decrease of GO concentration could not ensure the connectivity of Au electrodes by GO sheets.

**Figure 4:**
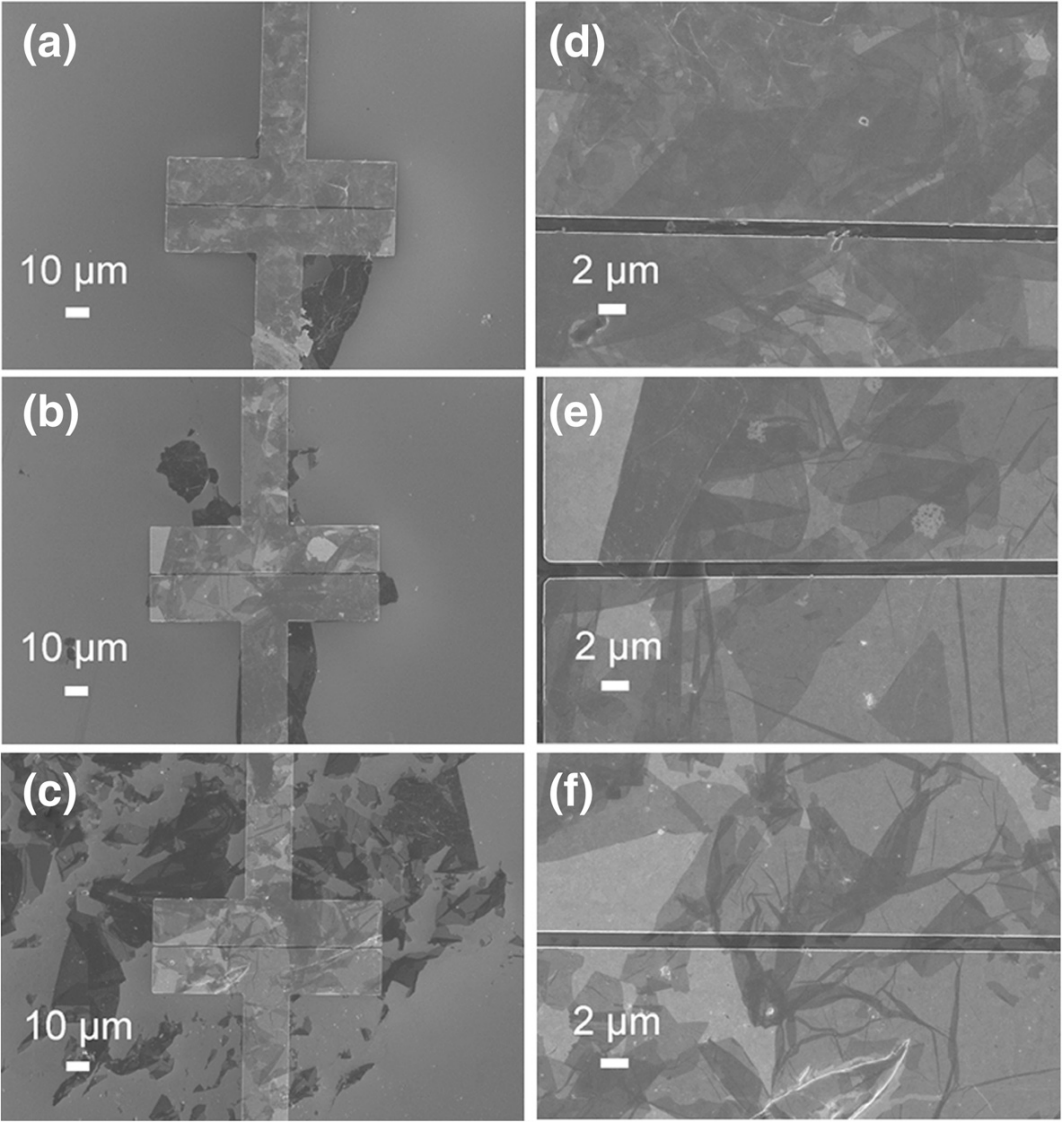
**SEM images of Hy-rGO bridged between Au electrodes self-assembled with different concentrations of GO. (a)** and **(d)** 1 mg/mL, **(b)** and **(e)** 0.5 mg/mL, and **(c)** and **(f)** 0.25 mg/mL.

After reduction of GO sheets on the electrodes by hydrazine, rGO bridged between Au electrodes was formed. As shown in Figure 4, all of the electrodes were covered with rGO sheets, which could ensure the electrical circuit be formed during the sensing detection. In addition, the number of rGO sheets decreased as the GO concentration decreases as well. Moreover, as for the GO concentration at 0.25 mg/mL, several rGO sheets were broken between the gaps of Au electrodes, which might be due to the strong surface tension during the reduction process, which might have a great effect on the sensing properties of the resultant rGO devices.

**Figure 5:**
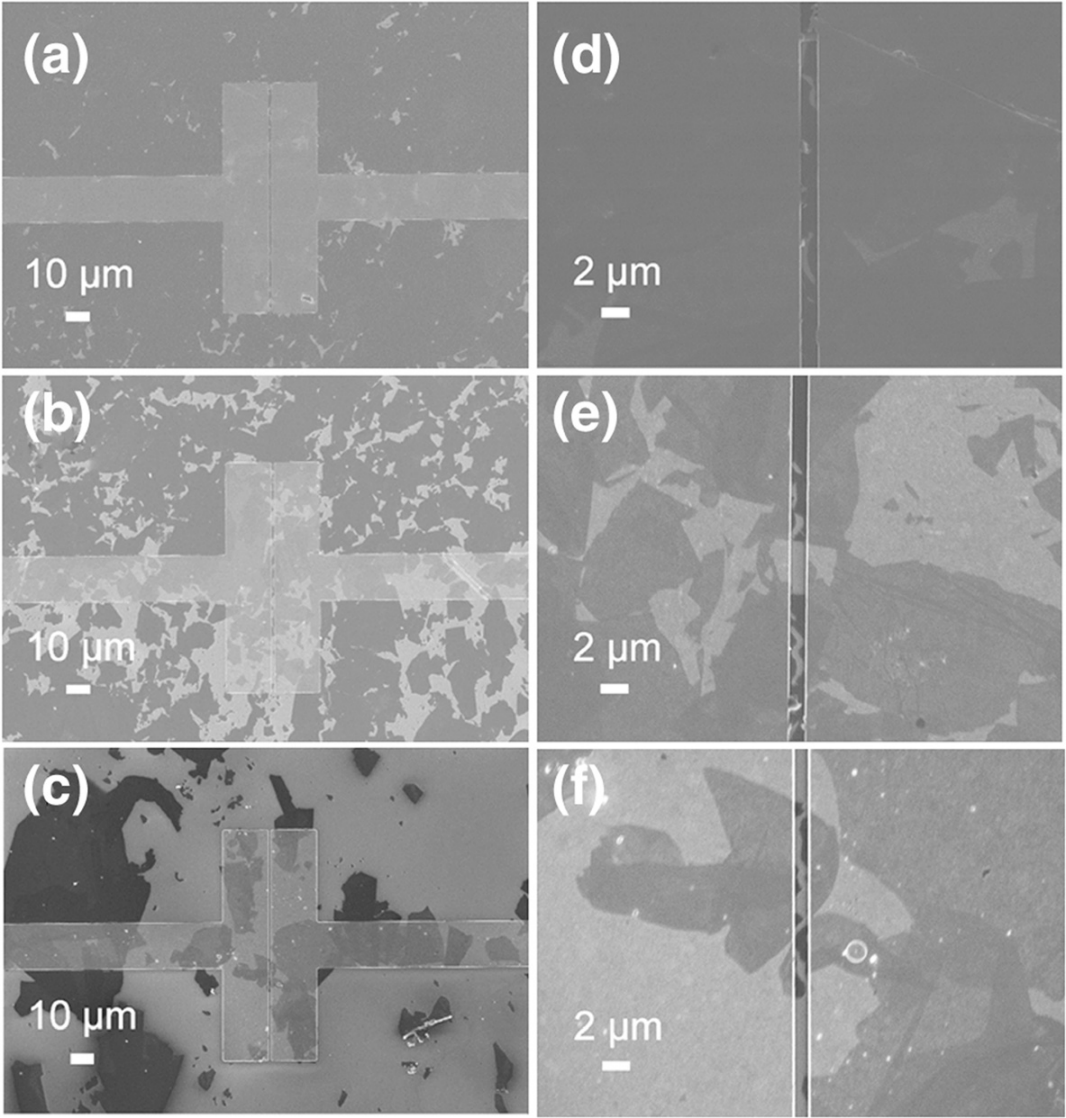
**SEM images of Py-rGO bridged between Au electrodes self-assembled with different concentration of GO. (a)** and **(d)** 1 mg/mL, **(b)** and **(e)** 0.5 mg/mL, and **(c)** and **(f)** 0.25 mg/mL.

The morphologies of Au electrodes assembled with Py-rGO have also been observed as shown in Figure 5. Similar with Hy-rGO, all of the electrodes were bridged by rGO sheets (as shown in Figure 5a, b, c, d, e, f). In addition, the enlarged images (as shown in Figure 5e, f) suggested that several GO sheets had been broken as well, and this phenomenon was much more severe when the GO concentration was as low as 0.25 mg/mL. Although this might affect the performance of the final devices, the connectivity of all of the electrodes by rGO sheets were fortunately achieved, which could be still used as sensing devices for gas detection.

**Figure 6:**
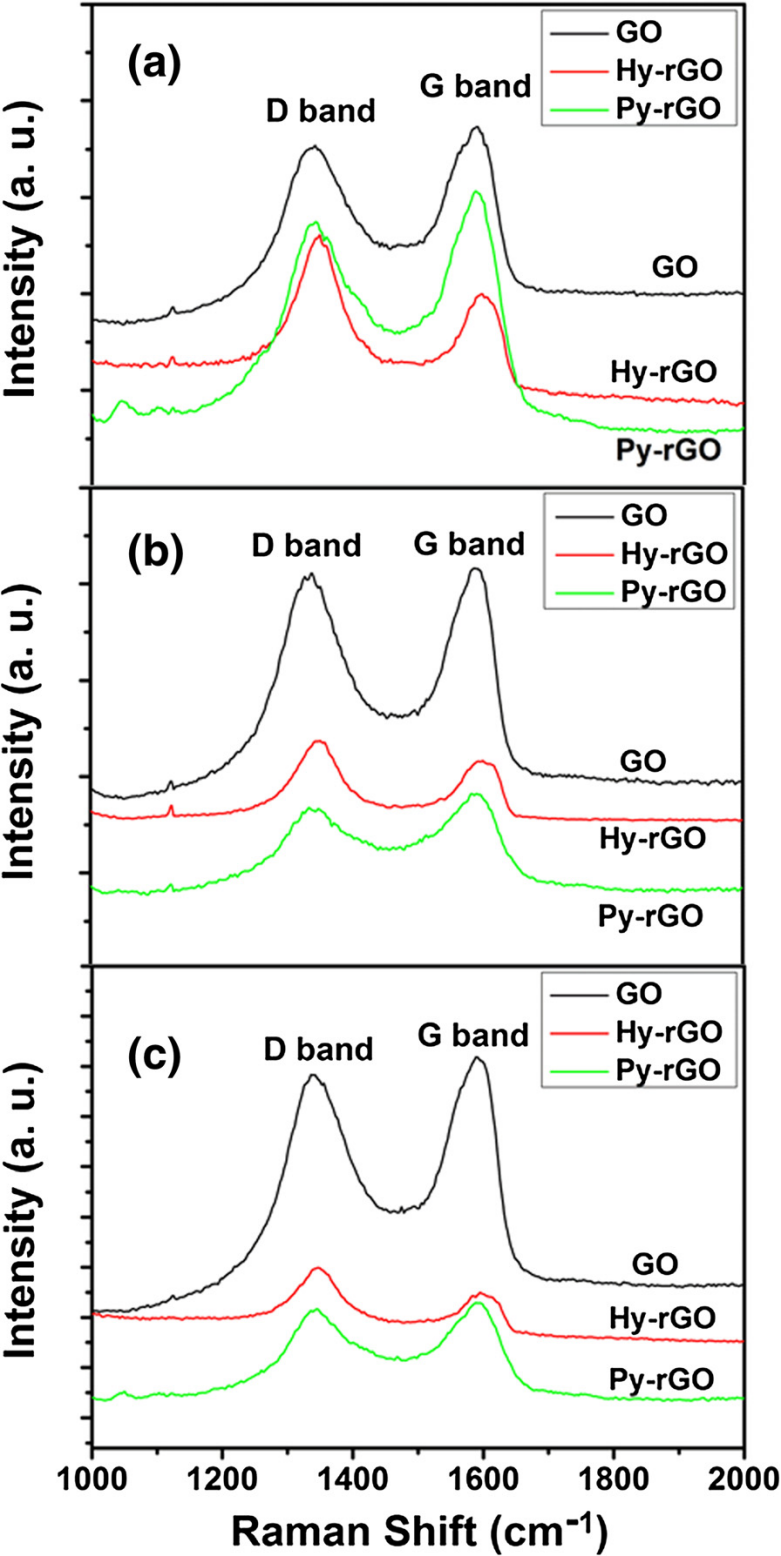
**Raman spectra of GO, Hy-rGO, and Py-rGO after assembly of the electrodes with GO concentrations. (a)** 1 mg/mL, **(b)** 0.5 mg/mL, and **(c)** 0.25 mg/mL with the excitation wavelength at 514 nm.

Raman spectroscopy is a powerful nondestructive tool to distinguish ordered and disordered crystal structure of carbon. Figure 6 exhibits the Raman spectra of GO, Hy-rGO, and Py-rGO after assembly of the electrodes with GO concentrations at (a) 1 mg/mL, (b) 0.5 mg/mL, and (c) 0.25 mg/mL with the excitation wavelength at 514 nm. All of spectra contained the following characteristic peaks: (1) the D band centered at 1,340 ~ 1,360 cm^-1^ (disorder mode), assigning to a breathing mode of κ-point phonons of A_1g_ symmetry; (2) G band located at 1,570 ~ 1,590 cm^-1^ (tangential mode), which was the E_2g_ phonon of C *sp*^2^ atoms. As for all of the GO concentrations, the characteristic peaks for assembled GO were similar, and the relative intensity of D band to G band was about 0.95. When GO sheets on the electrodes were reduced with hydrazine and pyrrole, the peaks of D and G bands of rGO blueshifted a little. Meanwhile, the relative intensity of D band increased substantially for Hy-rGO, i.e., an increase of D/G intensity ratio of rGO (about 1.40) compared to that of the GO could be observed. These changes suggested an increase in the average size of the *sp*^2^ domains upon reduction of GO, which agreed well with the Raman spectrum of the GO reduced by hydrazine that was reported by Stankovich et al. [42], indicating that reduction did happen. However, when GO was reduced by pyrrole, the situation was totally different. The peaks of D and G bands were wider than those of Hy-rGO, and the D/G intensity ratio decreased to about 0.90. This might be due to the polypyrrole (PPy) molecules adsorbed on the surfaces of rGO sheets. As we know, GO has long been recognized as having strong oxidizing properties, and it can serve as an oxidizing agent [43,44] for oxidative polymerization of pyrrole during the reduction process [45]. Since PPy molecule was a conducting polymer with ordered conjugated structures, PPy molecules on the surfaces of rGO sheets would decrease the D band (disordered structure) and meanwhile increase the G band (ordered structure) of rGO sheets. As a result, lower relative D band intensities were obtained.

In addition, the sizes of the crystalline domains within the rGO flakes could be estimated from the following equation [46]:

(1) $$L_{a}\;\left(\mathrm{nm}\right)=\left(2.4\times 10^{-10}\right)\lambda_{\mathrm{laser}}^{4}\;{\left(\frac{I_{D}}{I_{G}}\right)}^{-1}$$

where *L*_a_ is the size of the crystalline domains within CRG, λ_laser_ is the excitation wavelength of the Raman spectra, and $\frac{I_{D}}{I_{G}}$ is the *D*/*G* intensity ratio. A *D*/*G* ratio of 1.4 and 0.9 with the excitation wavelength at 514 nm for Hy-rGO and Py-rGO respectively in our work (Figure 3c) suggested that crystalline domains with the size of *ca*. 12 and *ca*. 18.7 nm respectively had been formed in within the resultant Hy-rGO and Py-rGO flakes.

### Evaluation of sensing devices based on assembled rGO sheets

**Figure 7:**
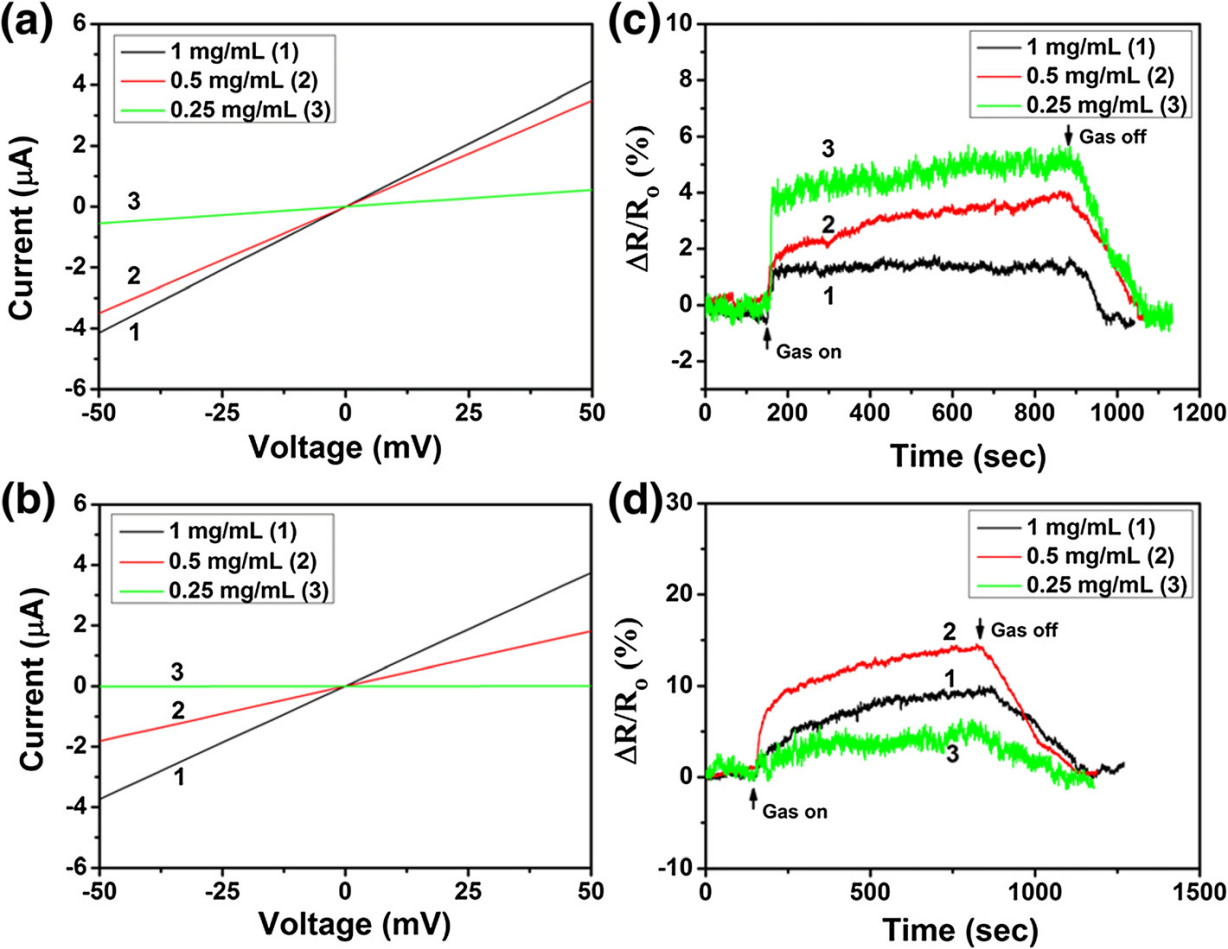
**The comparison of sensing properties of devices based on assembled rGO sheets.***I*-*V* curves of sensing devices based on Hy-rGO **(a)** and Py-rGO **(b)** fabricated with GO assembly concentration at 1, 0.5, and 0.25 mg/mL. Plot of normalized resistance change versus time for the sensing devices based on Hy-rGO **(c)** and Py-rGO **(d)** fabricated with GO assembly concentration at 1, 0.5, and 0.25 mg/mL (the concentration of NH_3_ gas is 50 ppm).

The resistances of the resultant sensing devices were measured by applying 50 mV of voltage and the results were shown in Figure 7a, b. The current versus voltage (*I*-*V*) curves of the sensing devices based on Hy-rGO and Py-rGO (as shown in Figure 7a, b), which were fabricated with GO assembly concentration at 1, 0.5, and 0.25 mg/mL, exhibited linear ohmic behaviors, suggesting that perfect circuits of the sensing devices had been achieved. In addition, the resistances of the sensing devices based on Hy-rGO and Py-rGO increased with the assembly concentration of GO solution. From Figure 7a, the resistances of Hy-rGO-based sensors could be calculated to be 12.3, 14.5, and 89.3 KΩ, respectively, when the assembly concentrations of GO were 1, 0.5, and 0.25 mg/mL. When the concentration was above 0.5 mg/mL, the resistances of the sensing devices had little changes. However, when the assembly concentration of GO solution decreased to 0.25 mg/mL, the resistance of the resultant device increased greatly. This might be due to the crack of the rGO sheets during the reduction process, which inevitably destroyed the electrical circuit of the device. Similar situations occurred for Py-rGO devices, as shown in Figure 7b, the resistances of the devices were 13.5 and 28.2 KΩ respectively when the assembly concentrations of GO solution were 1 and 0.5 mg/mL. Further decrease of GO concentration to 0.25 mg/mL resulted in rapid increase of resistance of the resultant Py-rGO device (8.3 MΩ). This value was much higher than the resistances of Hy-rGO-based devices. This might be ascribed to the following two reasons: (1) hydrazine was a stronger reducing agent during the reduction process, and as a result, the resistances of the resultant Hy-rGO devices were generally lower than those of Py-rGO devices, and this was also in agreement with the results as shown in Figure 7a,b; (2) much more cracks existed during the reduction process when pyrrole was used as a reducing agent. This could be proved by the SEM images as shown in Figure 5e,f; comparing with Hy-rGO devices (as shown in Figure 4e, f), much more cracks appeared, which had great effects on the final resistances of the resultant rGO devices.

NH_3_, a toxic gas, is very harmful to human health [47], and it is import to develop ammonia gas sensors and monitor for NH_3_ leaks. Hence, we used NH_3_ here as analyte in order to probe the sensing properties of the resultant Hy-rGO- and Py-rGO-based sensors. All of the sensors based on Hy-rGO and Py-rGO, which were fabricated with different assembly concentrations of GO solution, were tested toward 50 ppm NH_3_ balanced in synthetic air. The sensor response (*R*) toward NH_3_ gas was calculated according to the following equation:

(2) $$R\;\left(\%\right)=100\times \mathrm{\Delta R}/R_{0}=100\times \left(R_{\mathrm{gas}}-R_{0}\right)/R_{0}$$

where *R*_0_ is the resistance of rGO device before the exposure to NH_3_ gas, and *R*_gas_ is the resistance of rGO device in the NH_3_/air mixed gas [29].

Figure 7c, d displays the dynamic response of the resultant Hy-rGO- and Py-rGO-based sensing devices toward NH_3_ gas under the concentration of 50 ppm. In order to determine the optimal condition for the fabrication of sensing devices based on assembled rGO, the response of different sensing devices fabricated under different assembly concentration of GO solution were studied, and the exposure time of 12 min was defined here as the effective response time [29]. From Figure 7c,d, we can observe that the resistance of the devices increases significantly when NH_3_ was introduced into the chamber. As the assembly concentration of GO solution decreases, the response of the resultant Hy-rGO-based sensors increased from 1.6% to 5.3%, suggesting that fewer rGO sheets bridged in between the gaps of electrodes benefited for the final sensing performance of the sensing devices. Two main reasons may account for the decrease of sensing performance as the increase of GO concentration: (1) the large size of graphene sheets, which is different from the sheets reported before; the interconnecting point is much less and not good for the penetration of gas molecules, which causes the little variation of the resistance of the interior sheets; (2) the stacking structure of the graphene sheets with a dense structure can prevent the gas molecules from rapidly penetrating into the inner space of the films, which is different from the situation of graphene films with the porous or three-dimensional structure. This was also the case for Py-rGO-based sensors. When the assembly concentrations of GO solution was high (1 mg/mL), much more Py-rGO sheets were deposited on the surfaces of Au electrodes; as a result, it is hard for NH_3_ gas to penetrate into the rGO flakes and the complete interaction between NH_3_ and rGO sheets could not be ensured. Hence, a lower response value of 9.8% was obtained. When the assembly concentration of GO solution decreased to 0.5 mg/mL, the response of the resultant Py-rGO device increased to 14.2%, which was much higher than that of Py-rGO device fabricated with GO concentration at 1 mg/mL. However, further decrease of GO concentration did not increase the response of the resultant rGO sensing device. Instead, a much lower response value of 5.5% was obtained. This might be due to the crack of rGO sheets as mentioned above. The majority of rGO sheets were cracked between the electrode gaps, resulting in a rapid change of resistance of the resultant device and consequently leading to a lower response value. Most importantly, it was noticed that all of the responses of Py-rGO devices were higher than those of sensing devices based on Hy-rGO (as shown in Figure 7c,d), suggesting that Py-rGO-based sensing devices could be used as better sensors for the detection of NH_3_ gas. Since 0.5 mg/mL was the optimal parameter for the fabrication of the Py-rGO sensors, which exhibited the best sensing performance during the NH_3_ detection, further studies would focus on Py-rGO device fabricated under assembly concentration of GO solution at 0.5 mg/mL, with the purpose of demonstrating the potential utility as well as probing the sensing properties of the resultant assembled Py-rGO sensors.

**Figure 8:**
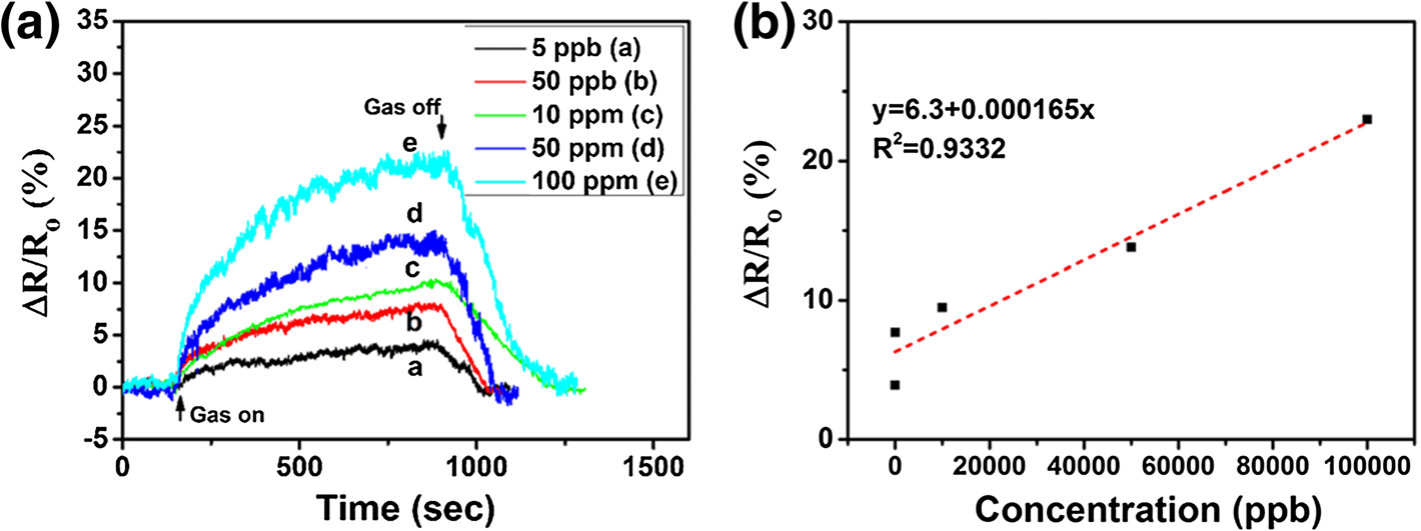
**The response performance of sensing devices based on assembled Py-rGO sheets. (a)** Plot of normalized resistance change versus time for the sensing device based on assembled Py-rGO upon exposure to NH_3_ gas with concentrations ranging from 5 ppb to 100 ppm: a, 5 ppb; b 50 ppb; c, 10 ppm; d, 50 ppm; and e, 100 ppm. **(b)** Relationship of response variation of the Py-rGO sensor as a function of NH_3_ concentration.

Various concentrations of NH_3_ gases, ranging from 5 to 100 ppm, were purged into the chamber in order to probe the sensing performance of the optimal Py-rGO sensor. As shown in Figure 8a, the plots of normalized resistance change versus time for the sensing device based on assembled Py-rGO upon exposure to NH_3_ gases with different concentrations were illustrated. The results revealed that the sensing device exhibited an excellent and highly reversible response to different concentrations of NH_3_ gases. When the NH_3_ gases were introduced into the chamber, the resistance of the sensing device increased significantly over a period of 12 min, and the increase of the concentration of NH_3_ gas can result in the increase of the resistance of the device, and all of the resistance variations can be distinctly observed when the devices expose to the NH_3_ gas with the concentration ranging from 5 ppb to 100 ppm. When the concentration of NH_3_ gas is 100 ppm, *ca*. 22% of the resistance change can be observed. As the concentration of NH_3_ gas decreases, the resistance change of the device decreases accordingly, and *ca*. 4.2% of the resistance change can be also observed when the concentration of NH_3_ gas was as low as 5 ppb. This is fascinating since the Py-rGO-based sensing devices exhibit much better response to NH_3_ gas than many other rGO-based devices [47,48]. Furthermore, the relationship of response variation of the Py-rGO sensor as a function of NH_3_ concentration has also been studied as shown in Figure 8b. The sensing signal changed linearly with the concentration of ammonia when the concentration is above 50 ppb. The linear relationship between the response of Py-rGO and the concentration of NH_3_ is in accordance with the work we reported before [29]. When the concentration is below 50 ppb, the sensing signal dropped rapidly (as shown in Figure 8b), which might be due to the PPy molecules covered on the surface of rGO sheets, and blocked the gas molecules interact with the rGO sheets, leading to a worse response to the NH_3_ gas molecules.

Furthermore, the sensor response exhibits an excellent recovery characteristic (as shown in Figure 8a). As illuminated with IR lamp together with flushed with dry air over the periods ranging from 134 to 310 s, the resistance of the device decreased and essentially recovered to the initial values. Since the Py-rGO sensors can be easily recovered, long-term practical work of the devices can be promised.

It is suggested that the excellent sensing properties of Py-rGO-based sensors are governed by the intrinsic properties of rGO as well as adsorbed PPy molecules. On one hand, rGO sheets still have parts of oxygen-based moieties and structure defects after chemical reduction process, which can generally lead to the p-type semiconducting behavior of the resultant rGO [29]. NH_3_, as a reducing agent, has a lone electron pair that can be easily donated to the p-type rGO sheets, leading to the increase of the resistance of the rGO devices. Since the rGO-based sensing devices studied in our work are fabricated by self-assembly technique, NH_3_ gas can interact with rGO sheets completely and result in excellent sensing performance of the devices during the testing process. On the other hand, PPy molecules, as conducting polymers, can be generally considered as excellent NH_3_ gas sensing materials. Hence, the PPy molecules, which are attached on the surfaces of rGO sheets, play important roles in the enhancement of the sensing performance of the rGO devices and consequently show a better sensing performance than that of Hy-rGO devices.

**Figure 9:**
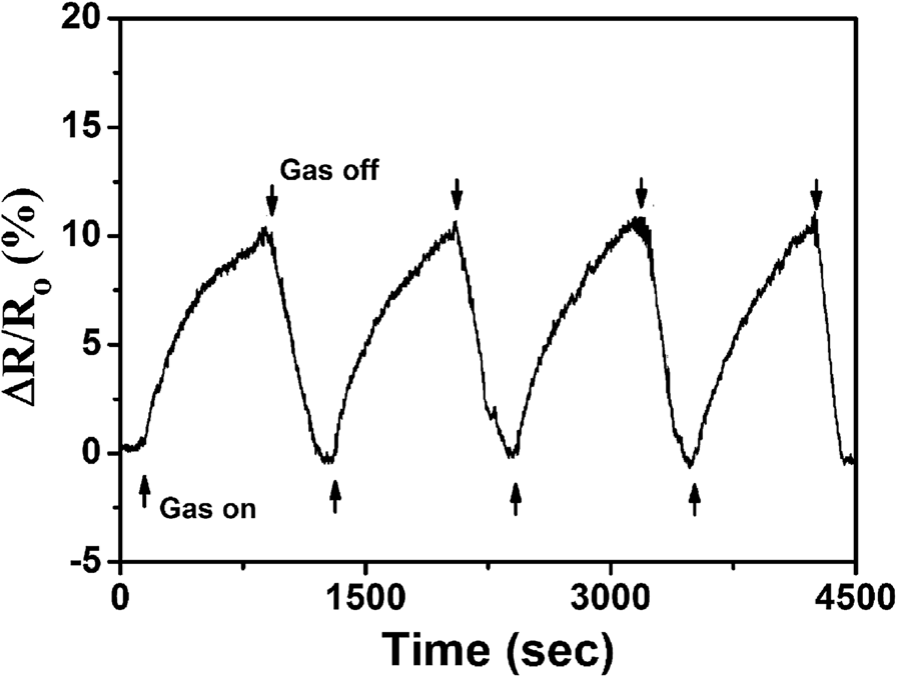
**The repeatability properties of the assembled Py-rGO sensor exposed to 10 ppm NH**_3_**.**

In addition, the repeatability of the Py-rGO sensing device has been studied as well. Figure 9 shows the relative resistance response of the assembled Py-rGO sensor as a function of time for detection of 10 ppm NH_3_ in four cycles, and the result suggests that the Py-rGO-based gas sensor exhibits a high reproducibility characteristic. Actually, the performance of the gas sensor based on Py-rGO is very stable for a long period time under normal operating conditions. Even after several months, the sensing device still shows excellent sensing performance. Therefore, it is suggested that sensors based on self-assembled Py-rGO can be considered as excellent sensing devices and have great potential in the sensing areas.

**Figure 10:**
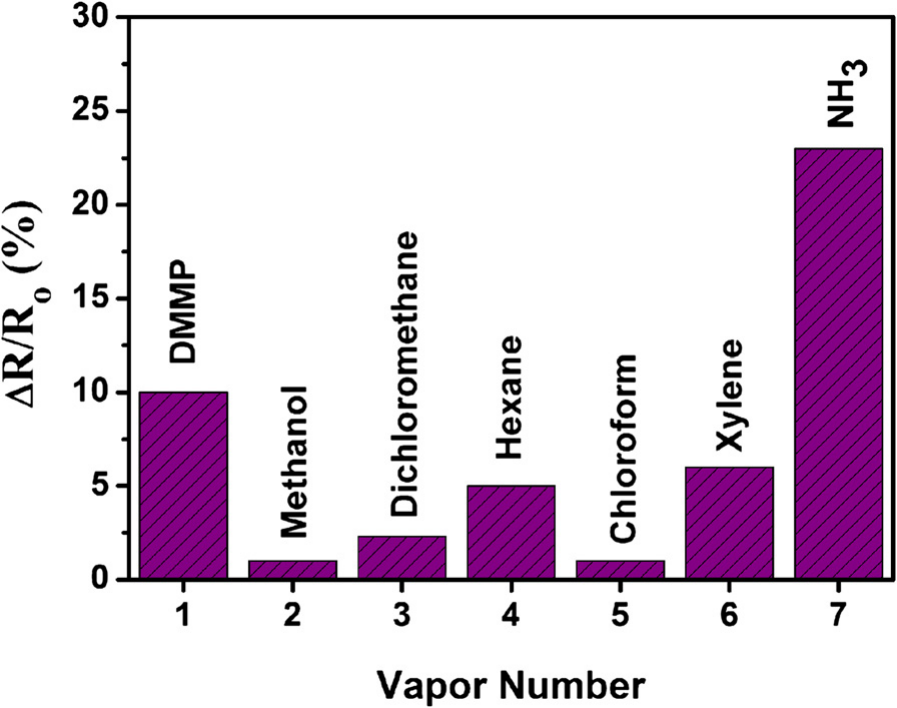
**Selectivity plot of the assembled Py-rGO sensing device.** Selectivity plot of the assembled Py-rGO sensing device exposed to 100 ppm NH_3_ compared with other analytes diluted to 1% of saturated vapor concentrations.

Finally, the selectivity of the assembled Py-rGO-based gas sensor, as another key factor for the evaluation of sensing devices, has also been studied (Figure 10). The responses of the sensor based on assembled Py-rGO sheets to 1% of saturated concentration of different analytes, e.g., DMMP, methanol, dichloromethane, hexane, chloroform, and xylene, have been studied and compared with the response of the device to 100 ppm NH_3_ gas. As shown in Figure 10, more than 2.3 times magnitude of response to 100 ppm NH_3_ gas for the Py-rGO sensor can be observed in comparison with other analytes. Since the concentration of NH_3_ gas is as low as 100 ppm while the concentrations of other analytes are much higher than that of NH_3_, it is suggested that the assembled Py-rGO-based sensor exhibits a high selectivity and can be considered as an excellent candidate for the detection of NH_3_ gas.

## Conclusions

In this work, a useful ammonia gas sensor based on chemically reduced graphene oxide (rGO) sheets using self-assembly technique has been successfully fabricated and studied for the first time. Negative GO sheets with large sizes (>10 μm) can be easily electrostatically attracted onto positive Au electrodes modified with cysteamine hydrochloride in aqueous solution. The assembled GO sheets on Au electrodes can be directly reduced into rGO sheets by hydrazine or pyrrole vapor and consequently provides the sensing devices based on self-assembled rGO sheets. The NH_3_ gas sensing performance of the devices based on rGO reduced from pyrrole (Py-rGO) have been investigated and compared with that of sensors based on rGO reduced from hydrazine (Hy-rGO). It is found that assembled Py-rGO exhibits much better (more than 2.7 times with the concentration of NH_3_ at 50 ppm) response to NH_3_ than that of assembled Hy-rGO. Furthermore, this novel gas sensor based on assembled Py-rGO showed excellent responsive repeatability to NH_3_. Since this technique can be incorporated with standard microfabrication process, we suggest that the work reported here is a significant step toward the real-world application of gas sensors based on self-assembled rGO.

## Competing interests

The authors declare that they have no competing interests.

## Authors' contributions

YYW has carried out the preparation of GO nanosheets, as well as fabrication of sensing devices. She has also performed all of analyses, except Raman characterization, and written the paper. NTH has also written the paper and got evolved in the preparation of samples. LLZ has dealt with fabrication and sensing test of sensors and carried out the analysis focusing on Raman characterization of samples. YW has participated in the AFM analysis and proof corrections. ZHZ have given some advices on the figure and text arrangement. YFZ, YHL, SS, and CSP have participated in the research guidance and paper correction. All authors read and approved the final manuscript.
